# Real-world treatment utilization in adults with chronic inflammatory demyelinating polyneuropathy in the United States

**DOI:** 10.3389/fneur.2025.1726857

**Published:** 2026-01-29

**Authors:** Cécile Blein, Chafic Karam, Clémence Arvin-Berod, Deborah Gelinas, Sergio Barrera-Sierra, Hashmath Ulla T. A. Syed, Charlotte Ward, Amit Goyal, Jeffrey Guptill

**Affiliations:** 1 argenx BVBA, Ghent, Belgium; 2 Department of Neurology, University of Pennsylvania, Philadelphia, PA, United States; 3 argenx US, Inc., Boston, MA, United States; 4 ZS Associates, Bengaluru, India; 5 ZS Associates, Bethesda, MD, United States; 6 ZS Associates, Princeton, NJ, United States

**Keywords:** chronic inflammatory demyelinating polyradiculoneuropathy, claims database, immunoglobulin, real-world evidence, treatment utilization

## Abstract

**Background:**

Management of chronic inflammatory demyelinating polyneuropathy (CIDP) is challenged by heterogeneity in severity, comorbidities, potential adverse effects, and treatment accessibility. This study aimed to elucidate treatment utilization among patients with CIDP in the United States (US) to identify potential unmet needs.

**Methods:**

Adult patients with CIDP were identified using Komodo Health’s Healthcare Map™ (January 2016–December 2020). Descriptive statistics related to utilization of CIDP treatments over 1-year post-index were analyzed. Among patients who used immunoglobulin (Ig), chronic Ig users were defined as patients with ≥8 Ig courses, and intermittent Ig users as patients with <8 Ig courses during 1-year post-index.

**Results:**

Among 3,409 patients with CIDP identified, the majority (81% [*n* = 2,758]) were treated for CIDP while 19% (*n* = 651) were untreated for CIDP during 1-year post-index. Steroids (73% [*n* = 2017]) followed by Ig (65% [*n* = 1,803]) were most commonly utilized. Of patients who used Ig, 62% (*n* = 1,113) were chronic and 38% (*n* = 690) were intermittent users during the 1-year post-index. A large proportion of Ig users received concomitant CIDP treatments, most commonly steroids. Patients who received >60 mg/day oral steroids on average over the 1-year post-index continued to use concomitant CIDP treatment, most commonly Ig.

**Conclusion:**

Steroids and Ig were mainstay treatments among patients with CIDP. A substantial proportion of Ig users were chronic users who also received other CIDP therapies, with steroids being most common. This suggests a potentially pronounced burden among patients treated with frequent Ig and steroids.

## 1 Introduction

Chronic inflammatory demyelinating polyneuropathy (CIDP) is a rare, immune-mediated disorder of the peripheral nervous system resulting in hallmark symptoms such as sensory loss, progressive muscular dysfunction, and fatigue (1–4). The prevalence of CIDP in the United States (US) is estimated at 8.9 per 100,000 persons, with the disease primarily affecting males between 40 and 60 years of age (1–3). Due to the overlapping clinical features present in other demyelinating neuropathies and no widely available diagnostic biomarker, diagnosis of CIDP can be challenging. Additional diagnostic challenges include the variable disease course experienced by patients, multiple CIDP subgroups, and complex clinical, electrodiagnostic, and laboratory diagnostic criteria. As a result, a substantial proportion of patients may be misdiagnosed, highlighting the need for a comprehensive approach to accurately diagnose and capture the true representation of disease burden in patients with CIDP (1, 4–6).

While CIDP lacks a definitive cure, the goals of treatment include alleviating symptoms, enhancing muscle strength, improving functional status, and maintaining long-term remission (7, 8). According to international treatment guidelines, first-line treatments include immunoglobulin (Ig), steroids, or plasma exchange (PLEX) (1, 3). Coupled with diagnostic challenges, treatment selection can be complex due to a variety of patient-specific factors that must be taken into consideration including disease severity, presence of comorbidities, potential adverse effects, cost, and access (4, 9). While some patients remain untreated, approximately 24% of patients with CIDP are refractory (defined as having failed at least 2 first-line treatments), with higher rates reported in some studies, and have clinical signs and symptoms despite available treatment options (10–12). Although intravenous and subcutaneous immunoglobulin (IVIg and SCIg) and corticosteroids remain the cornerstone of CIDP management, the treatment landscape is beginning to evolve. Subcutaneous efgartigimod alfa in combination with hyaluronidase (Vyvgart Hytrulo), a neonatal Fc receptor (FcRn) blocker, was recently approved by the US FDA in June 2024, for the management of CIDP (13–15). Other FcRn blockers, including batoclimab (NCT05581199) and nipocalimab (NCT05327114), are currently under clinical investigation for CIDP. In addition to FcRn blockade, other emerging immunomodulatory approaches are also being explored, such as targeting B-cells and/or plasma cells with anti-CD20 biologics and using proteasome inhibitors (PIs) to manage immune responses. Furthermore, empasiprubart, a complement C2 inhibitor, is also being evaluated for its potential role in the management of CIDP (NCT07091630).

Due to the multifaceted complexities associated with diagnosis, management, and treatment of CIDP, patients experience considerable clinical, economic, and humanistic burden. To date, however, little is known about the overall burden of CIDP, highlighting the need to further investigate its impact on patients’ lives. Compared to matched controls without CIDP, patients with CIDP have nearly a 3-fold higher risk of having comorbidities and higher rates of healthcare resource utilization including outpatient visits and hospitalizations (16). This may be due in part to many patients receiving immunosuppressive (e.g., with steroids) and/or immunomodulatory treatment (e.g., with Ig), both of which typically require infusions and can be associated with adverse events (AEs). While serious AEs from IVIg or SCIg, such as thromboembolic complications, are rare, common side effects may include headaches. Steroids can also lead to hypertension, the development of diabetes mellitus, glaucoma, depression, and a cushingoid appearance (3, 8, 17, 18). Additionally, economic burden may be significantly influenced by Ig utilization, which can account for up to 90% of drug costs (19). The clinical manifestations of CIDP, combined with treatment-related factors such as time spent receiving infusions, can adversely affect patients’ health-related quality of life. Furthermore, indirect costs arising from impaired productivity due to the condition and its treatment can further exacerbate the overall economic burden on patients and healthcare systems (20–22).

Large healthcare datasets, such as those based on insurance claims, offer the advantage of capturing a substantially larger and more heterogeneous patient population compared to clinical trials or single-center case studies, thereby enabling insights that reflect the variability observed in clinical practice. While such data sources are not without limitations, most notably, the reliance on diagnostic proxies and assumptions due to the absence of confirmatory clinical detail — they remain a valuable resource, particularly in the context of rare diseases such as CIDP, where evidence is limited. Although previous studies have examined real-world treatment utilization in CIDP (9, 23), many were constrained by small sample sizes or lacked representativeness and some existing data may be outdated.

Given these challenges, the present study was designed to identify a cohort of patients with a high probability of having true CIDP diagnoses, with the objective of generating evidence to enhance understanding of CIDP treatment utilization in clinical practice in the US. This study aims to address these knowledge gaps by offering a comprehensive analysis of treatment utilization within a larger, more representative cohort of patients with CIDP. The objective of this study was to isolate a robust population of patients with CIDP from a large US claims database to assess treatment utilization during the 1-year following the first observed diagnosis among patients with CIDP in the US.

## 2 Methods

### 2.1 Study design and data source

This retrospective cohort study utilized Komodo Health’s Healthcare Map™ claims database, which comprises comprehensive, longitudinal medical and pharmacy claims information from 150 payers across all geographic regions of the US (across Commercial, Medicare, Medicaid, self-insured; Table 1). The database is designed to minimize demographic bias across geography, payers, and race and ethnicity and is refreshed in near real time, providing the most up-to-date claims data (24). De-identified claims data, including patient demographic information, diagnoses, procedural and health service usage data, were obtained. International Classification of Disease (ICD)-9 and ICD-10 codes were used to identify diagnoses, National Drug Codes (NDC) were used to identify drugs of interest, and Current Procedural Terminology® (CPT) codes were used to identify diagnostic procedures. No identifiable or protected health information was obtained for use in this study.

**Table 1:** Baseline patient demographics and clinical characteristics.

Characteristic	Overall (*N* = 3,409)
Age, years, mean (SD)	59.4 (13.9)
Distribution by age, *n* (%)	
18–40	335 (10)
41–65	1,932 (57)
≥65	1,142 (33)
Gender, *n* (%)	
Male	2,055 (60)
Female	1,354 (40)
Race and ethnicity, *n* (%)	
Non-Hispanic Caucasian	1,405 (41)
Hispanic	292 (8)
Non-Hispanic African American	162 (5)
Non-Hispanic Asian	25 (1)
Other/unknown	1,525 (45)
Insurance type, *n* (%)^a^	
Commercial	1,680 (49)
Medicare	934 (27)
Medicaid	460 (13)
Other/multiple/unknown^b^	335 (10)
CCI, mean (SD)	2.0 (2.22)
Comorbidities, *n* (%)	
Any comorbidity	2,348 (69)
1	849 (25)
2	544 (16)
3+	955 (28)
Individual comorbidities, *n* (%)	
Diabetes without chronic complication	1,054 (31)
CPD^c^	753 (22)
Diabetes with chronic complication	720 (21)
Cerebrovascular disease	519 (15)
Peripheral vascular disease	514 (15)
CHF	341 (10)
Any malignancy^d^	313 (9)
Renal disease	282 (8)

a Percentages may not add up to 100% as patients may be tagged to multiple payer channels.

b Including self-insured, other/unknown, or dual-eligible.

c Including bronchitis, emphysema, asthma, chronic obstructive pulmonary disease, bronchiectasis, pneumoconiosis, and chronic drug-induced interstitial lung disorders.

d Including lymphoma and leukemia, except malignant neoplasm of skin.

CCI, Charlson Comorbidity Index; CPD, chronic pulmonary disease; CHF, congestive heart failure; SD, standard deviation.

### 2.2 Study population

**Figure 1:**
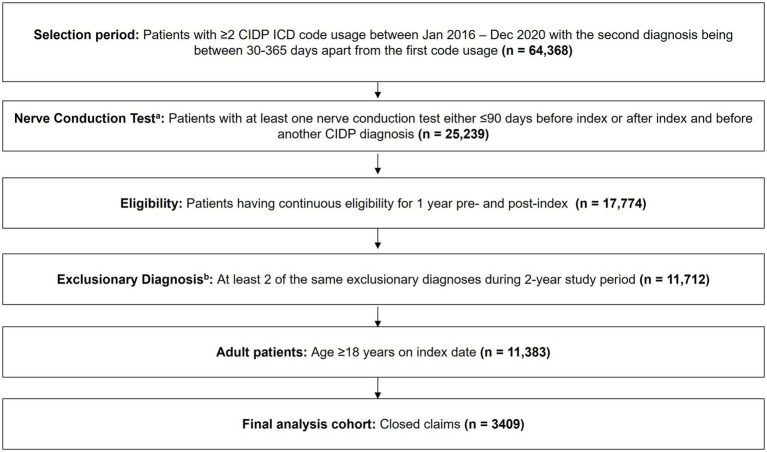
Patient selection. ^a^A nerve conduction test requirement was added to increase the robustness and certainty of identifying patients with CIDP. ^b^Exclusionary diagnosis includes amyloidosis, amyotrophic lateral sclerosis, autoimmune hemolytic anemia, B12 deficiency, celiac disease, chronic lymphocytic leukemia, dermatomyositis, fibromyalgia, Guillain-Barre syndrome, familiar neuropathy, human immunodeficiency virus, immune thrombocytopenic purpura, inclusion body myositis, bone marrow transplant, Kawasaki disease, multifocal motor neuropathy, multiple myeloma, multiple sclerosis, myasthenia gravis, necrotizing fasciitis, nonfamilial hypogammaglobulinemia, primary secondary immunodeficiency, sarcoidosis, organ transplant, systemic lupus erythematosus, toxic neuropathy, cancer chemotherapy. CIDP, chronic inflammatory demyelinating polyneuropathy; ICD, International Classification of Diseases.

Detailed inclusion criteria are summarized in Figure 1. Adults with at least 2 claims of CIDP diagnosis spanning the period of January 2016 to December 2020 were identified using ICD-9 or ICD-10 diagnosis codes (357.81 or G61.81). The cohort included a mixture of CIDP subtypes as more specific ICD codes are not available. Each patient’s first observed CIDP diagnosis identified during the study period was used as the index date. The second CIDP diagnosis was required between 30 and 365 days following the first observed CIDP diagnosis. To increase the certainty of correctly identifying true CIDP diagnoses while minimizing the inclusion of potentially misdiagnosed cases, an additional nerve conduction test (NCT) requirement was included. Patients were required to have undergone ≥1 NCT either ≤90 days before index or after index, and before another CIDP diagnosis (95,886, 95,885, 95,913, 95,911, 95,910, 95,912, 95,909, 95,861, 95,908, 95,887, 95,860, 95,863, 95,907, 95,864). This criterion was introduced as a result of expert consensus of CIDP-treating clinicians (6, 25). In addition, to meet the inclusion criteria, patients were required to have (1) continuous enrollment in their insurance plan for 1-year pre- and post-index to reduce the risk of missing data, and (2) have closed-claims data (data that is complete within the given study period). Importantly, given the challenges in correct CIDP diagnosis, a comprehensive list of exclusionary diagnoses was developed. Patients who had at least 2 claims of the same exclusionary diagnosis during the 1-year pre- and post-index were removed, as CIDP may have been a misdiagnosis. Given that the inclusion criteria required at least 2 CIDP diagnoses, the same approach was applied to exclusionary diagnoses (at least 2 diagnoses needed). Additional data regarding the list of exclusionary diagnoses can be found in Supplementary Table 1.

### 2.3 Study variables and outcomes

Baseline study variables assessed at index date included age, gender (female or male), race and ethnicity (Non-Hispanic Caucasian, Hispanic, Non-Hispanic African American, Non-Hispanic Asian, other/unknown), and insurance status (commercial, Medicare, Medicaid, other/multiple/unknown). Baseline variables assessed over the 1-year period prior to index were the Charlson Comorbidity Index (CCI) and baseline CIDP treatment utilization. CIDP treatments were defined as steroids (oral or IV), Ig (IVIg or SCIg), nonsteroidal immunosuppressant therapies (NSISTs; those that were included in this analysis were azathioprine, mycophenolate mofetil, methotrexate, cyclosporine, and tacrolimus), plasma exchange (PLEX), and biologic therapies (rituximab, inebilizumab, alemtuzumab, ocrelizumab, etanercept, interferon beta-1a, and natalizumab).

**Figure 2:**
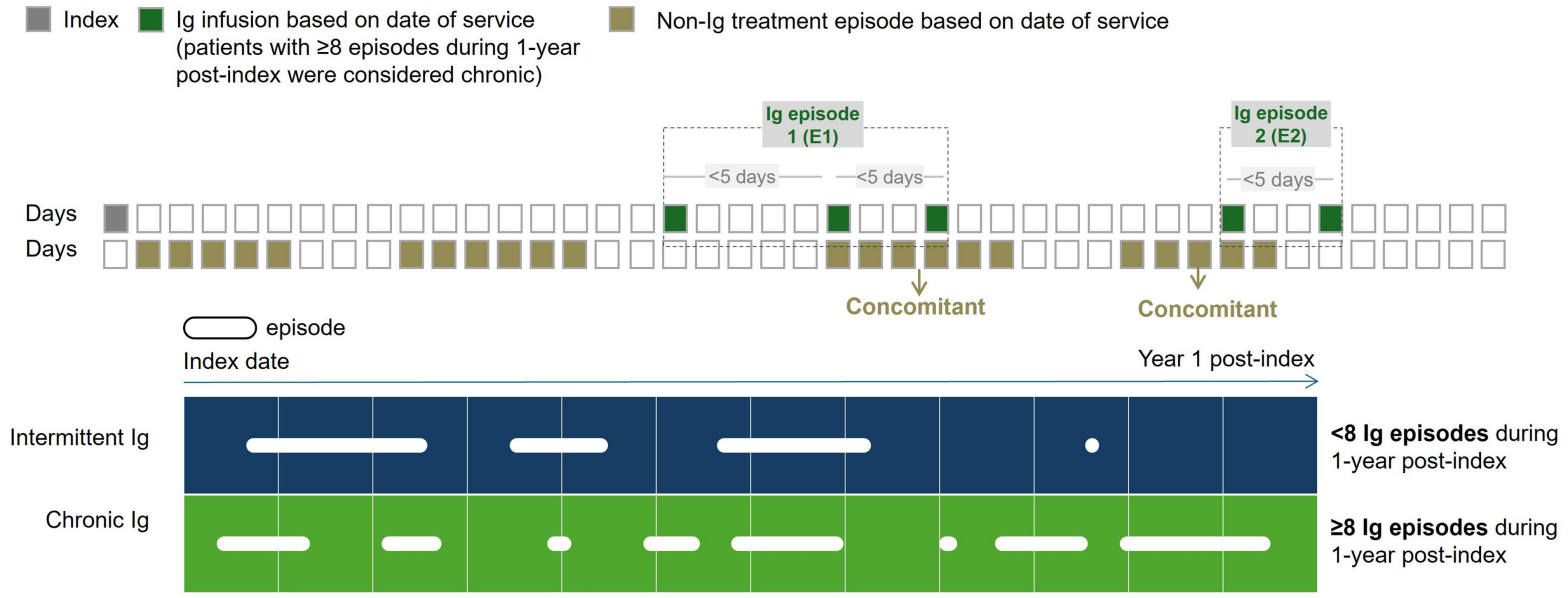
Illustration of Ig courses and classification of Ig users. Visual representation to illustrate the definitions associated with Ig utilization. Ig was defined as any claim indicating intravenous or subcutaneous Ig use. Squares represent days and the squares filled in with green represent an Ig infusion based on the date of service. The squared filled in brown illustrate concomitant treatments, defined as non-Ig treatments which were classified into courses based on date of service. Any courses that occurred concurrently with Ig courses were considered concomitant treatments. One Ig course was defined as a cluster of Ig infusions <5 days apart from one another. Patients were stratified into 2 cohorts based on the number of Ig courses during 1-year post-index. Circles represent an Ig course. Intermittent Ig users were defined as having <8 courses during 1-year post-index, whereas chronic Ig users were defined as having >8 courses during 1-year post-index. Ig, immunoglobulin.

The primary outcome was to assess the proportion of patients treated with different classes of CIDP-related treatments over 1-year post-index, in terms of number of claims, dosage, courses, and frequency. Treatment utilization was analyzed in the overall study population and within subgroups of patients to explore potential variations based on patient characteristics. Patients who received Ig (IVIg or SCIg) were categorized into sub-cohorts based on the frequency of Ig administration. An Ig course was defined as a cluster of Ig infusions occurring within 5 days of each other (Figure 2). Intermittent Ig users were defined as patients with <8 Ig courses for 1-year post-index, and chronic Ig users were defined as patients with ≥8 Ig courses during 1-year post-index, based on clinician input. Any utilization of other CIDP treatments that overlapped with Ig courses were considered concomitant treatments. Concomitant therapy was defined as any CIDP treatment that overlapped with an Ig episode/course with a minimum overlap of 2 days. Finally, patients among the overall cohort who received steroids were isolated to evaluate steroid dosage for 1-year post-index date.

### 2.4 Statistical analysis

Descriptive statistics were used to compare patient characteristics and CIDP-related treatments. Comparisons between chronic and intermittent Ig users were conducted using Welch’s t-test for continuous variables, and Chi-square tests for categorical variables. A *p* value <0.05 was considered statistically significant. All analyses were performed using Python 3.9.

## 3 Results

### 3.1 Patient demographic and baseline characteristics

A total of 3,409 patients were included in the final study cohort. Mean (SD) age was 59.4 (13.9) years, with over half aged between 41–65 years (57%, [*n* = 1,932]) and predominantly male (60%, [*n* = 2,055]). Patients identifying as non-Hispanic Caucasian (41% [*n* = 1,405]) compromised a majority of the study population (Table 1).

Commercial plans (49%, [*n* = 1,680]) and Medicare (27%, [*n* = 934]) were the most common types of health insurance. Overall, a substantial proportion (69% [*n* = 2,348]) had comorbidities, with 25% (*n* = 849) having at least one, 16% (*n* = 544) having two, and 28% (*n* = 955) having three or more comorbidities. Diabetes without chronic complication (31%, [*n* = 1,054]) and chronic pulmonary disease (22%, [*n* = 753]) were the most common comorbidities. Additional data regarding comorbidities can be found in Supplementary Table 2.

### 3.2 Treatment utilization

**Figure 3:**
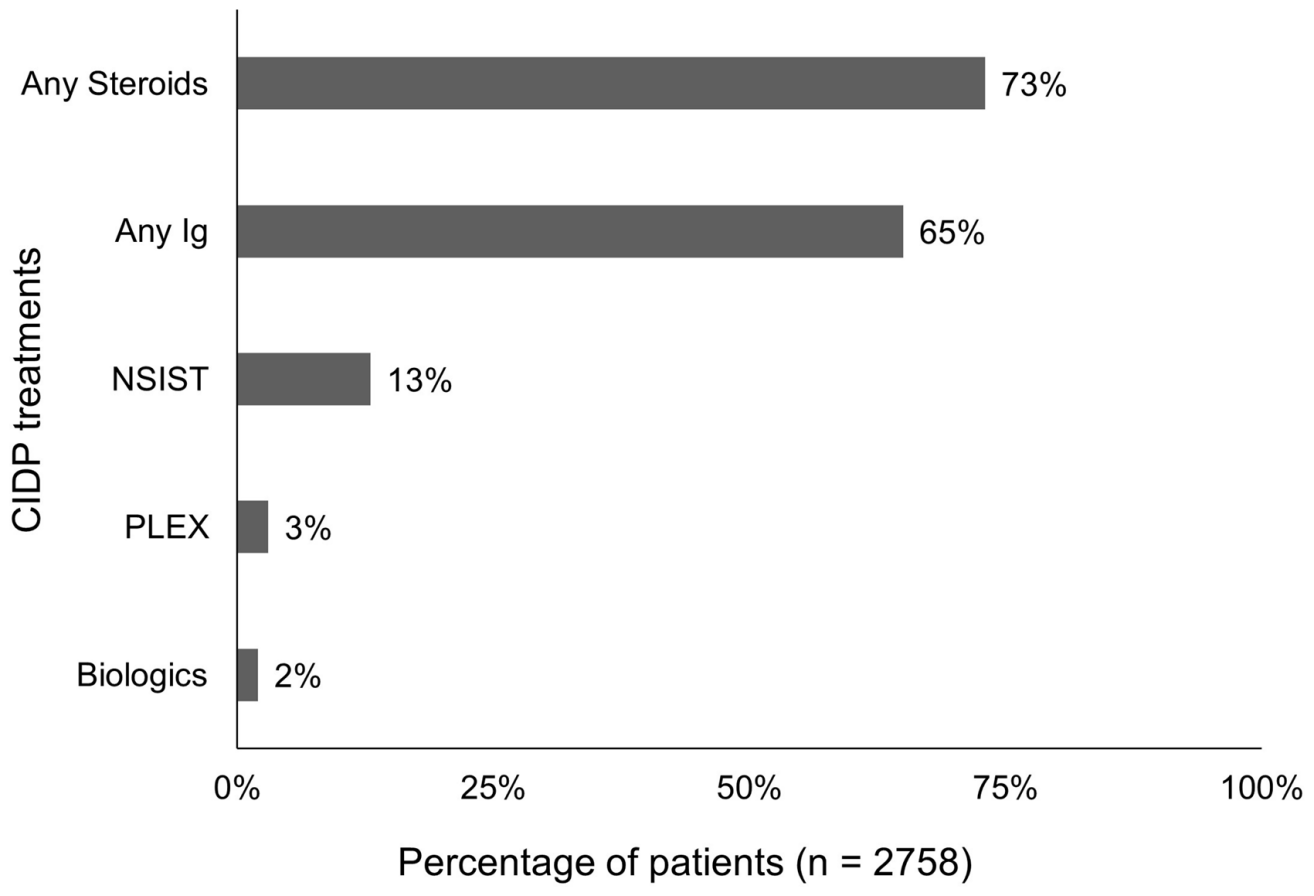
Proportion of patients who received CIDP treatments at least once during 1-year post-index. Bar graph illustrating the proportion of patients who received CIDP treatments at least once during 1-year post-index, noting the most common CIDP treatment were steroids, followed by Ig. CIDP, chronic inflammatory demyelinating polyneuropathy; Ig, immunoglobulin; NSIST; nonsteroidal immunosuppressive treatment; PLEX, plasma exchange.

Among the 3,409 patients with CIDP, the majority received treatment for CIDP during 1-year post-index (81% [*n* = 2,758]), while 19% (*n* = 651) were untreated. Steroids were the most common CIDP treatment used in the 1-year post-index period (used at least once by 73% [*n* = 2,017/2,758]) followed by Ig (used at least once by 65% [*n* = 1,803/2,758]; Figure 3). Among the 1803 patients receiving Ig, IVIg was used substantially more frequently than SCIg. Among the 2,017 patients who received steroids at least once, 60% (*n* = 1,204) used oral steroids, while 49% (*n* = 999) received IV steroids at least once. Of the 2,017 patients utilizing any steroids, 38% (*n* = 771/2,017) utilized steroids exclusively, while the majority used other CIDP treatments. Among the patients who received Ig treatment at least once during the 1-year post-index (*n* = 1803), the majority (72% [*n* = 1,293]) used Ig in combination with at least one other class of CIDP treatment, most commonly steroids (86% [*n* = 1,105]).

### 3.3 Ig utilization during 1-year post-index

Among the 1,803 patients who received Ig at least once in the 1-year post-index period, the majority were chronic Ig users (62% [*n* = 1,113]). Descriptive analyses demonstrated that chronic Ig users were more likely to receive concomitant CIDP therapies compared to intermittent Ig users (51% [*n* = 568/1,113] vs. 39% [*n* = 272/690]). Among Ig users, oral steroids were the most commonly used concomitant treatment, with their usage more prevalent among chronic Ig users than intermittent Ig users (32% [*n* = 354/1,113] vs. 20% [*n* = 140/690]), followed by IV steroids (29% [*n* = 322/1,113] vs. 22% [*n* = 152/690]; Table 2). A smaller proportion of chronic (10%; [*n* = 115/1,115]) and intermittent (6% [*n* = 40/690]) Ig users used concomitant NSISTs. Chronic Ig users had an average of 14.3 Ig courses over 1-year post-index, contrasting with 3.6 courses for intermittent Ig users. Additional data regarding the distribution of Ig courses for chronic and intermittent Ig users can be found in Supplementary Figure 1.

**Table 2:** Overview of Ig use and concomitant treatments (Ig utilization during 1-year post-index).

Ig utilization	Chronic Ig^a^ (*n* = 1,113)	Intermittent Ig^b^ (*n* = 690)	*p* value
Patients with ≥1 Ig course with concomitant treatment,^c^ *n* (%)	568 (51)	272 (39)	1.98 E-80^d^
Average number of Ig courses per patient	14.3	3.6	
Median time between Ig courses, days	20	27	2.03 E-34^e^
Patients with at least 1 concomitant treatment with Ig			
Oral steroids	354 (32)	140 (20)	2.08 E-90^d^
IV steroids	322 (29)	152 (22)	3.42 E-69^d^
NSIST	115 (10)	40 (6)	4.46 E-73^d^
PLEX	5 (0)	1 (0)	7.57 E-61^d^

a Chronic users are patients with ≥8 Ig courses in the 1-year follow-up.

b Intermittent users are patients with 1–7 Ig courses in the 1-year follow-up.

c Concomitant treatments are defined as non-IVIg treatments initiated and concomitant (based on date of service) from initiation of IVIg course to the beginning of the next IVIg course.

d Chi-square test was used for categorical variables. A *p* value <0.05 was considered statistically significant.

e Welch, s *t*-test was used for continuous variables. A *p* value <0.05 was considered statistically significant.

Ig, immunoglobulin; NSIST; nonsteroidal immunosuppressive treatment; PLEX, plasma exchange.

### 3.4 Steroid utilization during 1-year post-index

Among the 2,017 patients who utilized steroids during 1-year post-index, the majority (60%; [*n* = 1,204]) received oral steroids and 49% (*n* = 999) received IV steroids at least once. Among the oral steroid users (*n* = 1,204), a total of 1,007 patients had steroid dosing information available. Patients were categorized according to their average daily steroid dose across 1-year post-index in order to evaluate any differences in treatment utilization (Table 3). Across all steroid dosing categories, time on oral steroid treatment was roughly 3 months on average, with 32% (*n* = 320/1,007) of patients receiving greater than 60 mg of steroids on average per day. A significant proportion of steroid users receiving greater than 60 mg per day continued to use concomitant treatments, most commonly Ig (Table 3).

**Table 3:** Oral steroid usage in patients with dosage information available (steroid utilization during 1-year post-index).

Oral steroid usage	Overall *n* = 1,007 (100%)^a^^,^^b^	0–30 mg/day *n* = 352 (35%)	>30–60 mg/day *n* = 335 (33%)	>60 mg/day *n* = 320 (32%)
Annualized time on treatment, days				
Mean (SD)	89 (105)	113 (118)	75 (95)	81 (95)
Median (IQR)	35 (10–150)	60 (14–202)	30 (9–108)	30 (8–122)
Average daily dose, mean (SD), mg/day	60 (74)	17 (8)	45 (9)	122 (104)
Patients with usage of other treatments, *n* (%)				
Ig	581 (58)	185 (53)	210 (63)	186 (58)
NSIST	198 (20)	78 (22)	63 (19)	57 (18)
Biologics	26 (3)	9 (3)	8 (2)	9 (3)
PLEX	31 (3)	8 (2)	12 (4)	11 (3)

a Dosages are standardized based on prednisolone.

b Oral steroid patients were included if dosing information was available and could be calculated.

Ig, immunoglobulin; IQR, interquartile range; IVIg, intravenous Ig; NSIST; nonsteroidal immunosuppressive treatment; PLEX, plasma exchange; SD, standard deviation.

## 4 Discussion

This study investigated treatment utilization during the 1-year period following the first observed diagnosis among patients with CIDP, based on US claims data. The mainstay treatments for CIDP in the US were steroids, followed by Ig. Among patients who received Ig, a substantial proportion were chronic Ig users who received concomitant treatments, with steroids being the most common. Given the high steroid and Ig use in this patient population, the treatment burden is substantial and represents a potential unmet need to be addressed.

The complexity of CIDP diagnosis in clinical practice makes it inherently challenging to capture a large cohort of patients with confirmatory diagnoses using claims data (26). Expert consensus indicates that an NCT is essential to confirming a diagnosis of CIDP; therefore, patients with CIDP diagnoses but without an NCT claim identified were excluded from our analysis. While still imperfect, the exclusion of approximately two-thirds of patients with at least 2 CIDP diagnoses upon the addition of the NCT requirement in our study underscores the challenges of isolating a reliable CIDP population from claims data. Continuing to think beyond traditional methods of patient selection that solely rely on disease codes is required to increase accuracy and specificity to provide meaningful evidence. Incorporating additional targeted inclusion criteria in the future based on clinical input may help to better capture patients with CIDP and assess the true representation of their burden beyond isolated cohorts.

Our results showed that 73% of patients with CIDP received steroids at least once, a large proportion of whom received greater than 60 mg/day of oral steroids. These findings are consistent with results from a previous study which noted up to 55.2% of patients with CIDP received steroids (9, 18). We recognize that steroids may have been prescribed for different reasons, including to treat (1) CIDP symptoms themselves muscle weakness and sensory deficits can be treated with steroids according to EFNS treatment guidelines (1), (2) Ig side effects, or (3) comorbidities. However, the precise reasons behind each prescription were not available in this dataset (or in claims databases in general). Although steroids are also often used to ameliorate infusion-related AEs that may be related to Ig use or to decrease the need for frequent IVIg infusions, our study findings indicated that 38% of patients utilized steroids exclusively. In clinical practice, steroids may be an attractive treatment option since they are easy to administer, often inexpensive, and demonstrate short term efficacy (9, 18). While steroids may provide clinical benefit in the management of patients with CIDP, there are concerns of increased risk of AEs associated with both the dose and duration of their long-term use (27). Mild to moderate AEs have been reported in patients with CIDP following treatment with steroids and IVIg treatments (28, 29). Therefore, clinicians need to consider that exposure of patients with CIDP to high dose steroid treatments may exacerbate the risk of potentially serious AEs. Whether and how treatment decision-making is impacted by CIDP symptom severity, comorbidities, costs, or other specific reasons could not be assessed in this study and warrant future studies to delineate.

Our study revealed Ig was the second most common treatment received by patients with CIDP (65%), despite being considered the standard of care and recommended as first-line therapy in the international treatment guidelines on CIDP management (1). While evidence suggests most patients who benefit from Ig treatment demonstrate a favorable response within the first 24 weeks of treatment (30), there are no good predictors of Ig treatment response. As a result, a subset of patients may experience limited treatment efficacy, needing concomitant treatments to provide sufficient symptom control, or early discontinuation (30). Reflecting this evidence, the international treatment guidelines recommend that Ig treatment response be evaluated after a few months, including tapering Ig dosing based on improvement or lack of response to the loading dose (1). Studies suggest that IVIg has been associated with serious adverse effects, such as renal impairment, thrombosis, arrhythmia, aseptic meningitis, hemolytic anemia, and transfusion-related acute lung injury (31). The majority of patients who received Ig treatment at least once during 1-year post-index utilized Ig in combination with an additional class of CIDP treatment, which was particularly evident in chronic Ig users. Additionally, more than half of the patients who received greater than 60 mg/day of oral steroids on average used concomitant CIDP treatments, most commonly Ig. While these results could suggest that a subset of patients may experience inadequate control of CIDP-related symptoms with available treatments, it is important to note that guidelines generally do not recommend combining therapies in most patients. In the current study, the difference between true combination therapy and the use of added steroids for preventing AEs could not be established. Therefore, further research is warranted to understand the rationale behind certain treatment decisions.

Given our study findings revealing a substantial proportion of patients with CIDP using frequent Ig and prolonged therapeutic dosing of steroids, treatment burden for patients with CIDP may be currently underestimated. Even with chronic Ig use, steroids may be needed as a bridge therapy to manage CIDP symptoms between Ig courses. However, as the rationales associated with treatment decisions were not available in the dataset, further investigation is required to clarify how these practices correspond to clinical reasons, as well as guideline adherence. Although our study findings show a high treatment burden in CIDP, particularly with long-term use of steroids and Ig, we recognize that claims data do not provide enough clinical detail to determine whether all treatments were appropriate. The possibility of overtreatment, especially in patients with stable disease or diagnostic uncertainty, cannot be assessed and remains an important consideration. This may represent a different kind of unmet need in CIDP care and highlights the importance of further studies using clinically detailed data sources.

### 4.1 Limitations and future trends

Claims-based retrospective analyses provide valuable insights into treatment utilization across large and diverse patient populations over time but have inherent limitations. Consistent with limitations in previous US-claims-based retrospective studies, clinical details including diagnostic workups, disease severity, symptom progression, reasons for treatment switching or initiation, as well as key CIDP clinical outcome measures (such as PROs/CROs) were not captured, which constrained interpretation of clinical reasons behind descriptive treatment patterns. As widely known, CIDP remains a clinically heterogeneous and diagnostically complex condition, and although we applied robust identification criteria to reduce misdiagnosis, the absence of confirmatory clinical data and the evolving diagnostic criteria over the study period (2016–2020) may contribute to some diagnostic uncertainty. Moreover, since specific ICD codes for each CIDP subtype do not exist, we were not able to ascertain how treatments may differ by subtype, which should be investigated in a future study. As our study was limited to the 1-year following index diagnosis, additional research on the long-term impact of chronic usage of Ig and high dose steroids on health outcomes needs to be further explored. For a substantial number of Ig products identified in claims, the route of administration (IV or SC) was unclear; therefore, the study is limited in providing a clear delineation of the distribution for IV or SC Ig usage in CIDP. Furthermore, a short tapering period when switching from steroids to Ig (e.g., a few days of overlap) may be classified as concomitant use rather than a treatment switch based on the study’s overlap criteria, potentially leading to an overestimation of concomitant therapy and underrepresentation of true switching patterns. Future studies investigating practice patterns among clinicians may help elucidate clinical unmet needs associated with the treatment and management of patients with CIDP.

## 5 Conclusion

Our study focused on evaluating treatment utilization in patients with CIDP in the US, based on a robust claims-based dataset. Results highlighted patients predominantly received steroids and Ig as mainstay therapies during the 1-year period following the first observed CIDP diagnosis. Moreover, a significant proportion of patients who received Ig in the 1-year post-index period were chronic Ig users, with a substantial proportion receiving other concomitant CIDP therapies. Similarly, a significant proportion of steroid users utilized high dose steroids with concomitant treatments, underscoring a potential unmet need for safe and effective therapies that can reduce treatment burden and long-term risks of developing AEs. Additional research, including identifying clinical and economic unmet needs, is essential in order to optimize health outcomes in patients with CIDP.

## Data Availability

The original contributions presented in the study are included in the article; further inquiries can be directed to the corresponding author.
