# Supplementary material for: Real-world treatment utilization in adults with chronic inflammatory demyelinating polyneuropathy in the United States
Source: Front Neurol. 2026 Jan 29;16:1726857. doi: 10.3389/fneur.2025.1726857 (PMC12896209; doi:10.3389/fneur.2025.1726857)
Supplement: Supplementary file 1 [file Supplementary_file_1.docx]

## **Supplementary Appendix**

Supplementary Figure 1: Distribution of patients based on the number of Ig episodes.

Chronic Ig users are patients with ≥8 Ig episodes during 1-year post-index. Intermittent Ig users are patients with <8 Ig episodes in the 1-year post-index period. Bar graph illustrating the distribution of Ig episodes during 1-year post-index.

Supplementary Table 1: List of exclusionary diagnoses.

| **List of exclusionary diagnoses** |
| --- |
| Amyloidosis |
| Amyotrophic lateral sclerosis |
| Autoimmune hemolytic anemia |
| B12 deficiency |
| Celiac disease |
| Chronic lymphocytic leukemia |
| Dermatomyositis |
| Fibromyalgia |
| Guillain-Barre syndrome |
| Familial neuropathy |
| Human immunodeficiency virus |
| Immune thrombocytopenic purpura |
| Inclusion body myositis |
| Bone marrow transplant |
| Kawasaki disease |
| Multifocal motor neuropathy |
| Multiple myeloma |
| Multiple sclerosis |
| Myasthenia gravis |
| Necrotizing fasciitis |
| Nonfamilial hypogammaglobulinemia |
| Primary secondary immunodeficiency |
| Sarcoidosis |
| Organ transplant |
| Systemic lupus erythematosus |
| Toxic neuropathy |
| Cancer chemotherapy |

Supplementary Table 2: Common comorbidities in each category.

|  | Overall (*N* = 3409) |
| --- | --- |
| **1 comorbidity** | **849 (100)** |
| Chronic pulmonary disease^a^ | 211 (25) |
| Diabetes without chronic complication | 143 (17) |
| Peripheral vascular disease | 91 (11) |
| **2 comorbidities** | **544 (100)** |
| Diabetes with chronic complication & diabetes without chronic complication | 141 (26) |
| Chronic pulmonary diseasea & diabetes without chronic complication | 30 (6) |
| Cerebrovascular disease & diabetes without chronic complication | 25 (5) |
| **3+ comorbidities** | **955 (100)** |
| Diabetes with chronic complication & diabetes without chronic complication & peripheral vascular disease | 28 (3) |
| Diabetes with chronic complication & diabetes without chronic complication & renal disease | 25 (3) |
| Diabetes with chronic complication & diabetes without chronic complication & chronic pulmonary diseasea | 22 (2) |

Data are expressed as *n* (%).

^a^Including bronchitis, emphysema, asthma, chronic obstructive pulmonary disease, bronchiectasis, pneumoconiosis, and chronic drug-induced interstitial lung disorders.
